# Syndecan-4 Regulates Muscle Differentiation and Is Internalized from the Plasma Membrane during Myogenesis

**DOI:** 10.1371/journal.pone.0129288

**Published:** 2015-06-12

**Authors:** Sissel B. Rønning, Cathrine R. Carlson, Espen Stang, Svein O. Kolset, Kristin Hollung, Mona E. Pedersen

**Affiliations:** 1 Nofima AS, Pb 210, NO-1431 Ås, Norway; 2 Institute for Experimental Medical Research, Oslo University Hospital and University of Oslo, Oslo, Norway; 3 KG Jebsen Cardiac Research Center and Center for Heart Failure Research, University of Oslo, Oslo, Norway; 4 Department of Pathology, Oslo University Hospital, Rikshospitalet, P.O. Box 4950 Nydalen, 0424 Oslo, Norway; 5 Department of Nutrition, Institute of Basic Medical Sciences, University of Oslo, Oslo, Norway; University of Minnesota Medical School, UNITED STATES

## Abstract

The cell surface proteoglycan syndecan-4 has been reported to be crucial for muscle differentiation, but the molecular mechanisms still remain to be fully understood. During *in vitro* differentiation of bovine muscle cells immunocytochemical analyses showed strong labelling of syndecan-4 intracellularly, in close proximity with Golgi structures, in membranes of intracellular vesicles and finally, in the nuclear area including the nuclear envelope. Chase experiments showed that syndecan-4 was internalized from the plasma membrane during this process. Furthermore, when syndecan-4 was knocked down by siRNA more myotubes were formed, and the expression of myogenic transcription factors, β1-integrin and actin was influenced. However, when bovine muscle cells were treated with a cell-penetrating peptide containing the cytoplasmic region of syndecan-4, myoblast fusion and thus myotube formation was blocked, both in normal cells and in syndecan-4 knock down cells. Altogether this suggests that the cytoplasmic domain of syndecan-4 is important in regulation of myogenesis. The internalization of syndecan-4 from the plasma membrane during muscle differentiation and the nuclear localization of syndecan-4 in differentiated muscle cells may be part of this regulation, and is a novel aspect of syndecan biology which merits further studies.

## Introduction

Growth of adult muscle occurs through activation and fusion of myogenic satellite cells with existing muscle fibres. The muscle stem cells are quiescent, but will upon injury, disease or exercise undergo myogenesis which leads to the formation of more muscle tissue. The conversion of mononuclear muscle precursors (myoblasts) into multinucleated myotubes is a complex process and is still not fully characterized. The activation of muscle satellite cells are characterized by the rapid expression of myogenic regulatory transcription factors (MRFs), in response to growth factors and transduction of signals into the cells via cell surface localized receptors, such as the fibroblast growth factor dependent receptor tyrosine kinase (FGFR). The interactions of FGFR with proteoglycans (PGs) have been shown to enhance activation of receptor-mediated signalling [1]. The PGs are highly sulphated macromolecules, whose protein cores carry covalently attached carbohydrate chains named glycosaminoglycans (GAGs). The GAG chains on the protein core are unbranched polysaccharide chains composed of repeating disaccharide units [2]. Cell surface PGs are responsible for recruiting soluble growth factors, allowing them to bind to their respective receptors [1]. The majority of cell surface PGs is represented by syndecans and glypicans. The syndecan family consists of four PGs: syndecan-1,-2,-3, and -4, which all are transmembrane molecules [3, 4], carrying mostly heparan sulfhate (HS) chains. Glypicans are also HSPGs, but are anchored by a glycosylphosphatidyl inositol moiety to the outer membrane leaflet. Glypicans and syndecans commonly coexist on cell surfaces, but can also be enriched in different plasma membrane domains [4]. Syndecans are characterized by a conserved transmembrane domain, a short cytoplasmic domain with two conserved regions (C1 and C2) flanking a unique variable domain (V-region) which differ between each syndecan, and a large diverse extracellular domain with specific GAG attachment sites. Syndecans typically respond to binding of extracellular ligands, but compared to other cell surface localized receptors (e.g. growth factor receptors) syndecans have additional, unique characteristics. They can interact, through the GAG chains, with several different extracellular ligands, with a much higher number of ligands bound per syndecan molecule compared to growth factor receptors. Syndecans have important functions in processes like cell adhesion, immunological reactions and regulation of growth and cellular morphology due to their ability to bind growth factors, cytokines, chemokines, morphogenes, extracellular matrix proteins, cell-cell adhesion receptors and cytoskeletal proteins, mediated through the GAG chains or the core proteins [5].

Syndecans are reported to have crucial functions in muscle development, maintenance and regeneration, and the syndecan involvement in muscle development has previously been investigated in turkey, mice and *Drosophila* [6, 7]. Syndecan-3 and -4 are the only syndecans expressed in regenerating muscle, and they have distinct, yet essential roles in muscle development and regeneration [8]. Cornelison *et al*. showed that syndecan-4^-/-^ mice were unable to regenerate damaged muscle, and displayed deficient satellite cell activation, proliferation, MyoD expression and differentiation [8]. The cytoplasmic domain is involved in syndecan-4 oligomerization and cytoskeletal organization [9–11], PKCα localization and activation [5, 12], and cell migration [13]; all processes essential for myogenesis.

Although syndecan-4 has been reported to be crucial for muscle differentiation, the molecular mechanisms remain to be fully determined. In the present study we aimed to investigate and characterize the importance of syndecan-4 during bovine muscle differentiation *in vitro*. Our data show that syndecan-4 is internalized from the plasma membrane via the endocytic pathway, and localizes to the nuclear area including the nuclear envelope during muscle differentiation. Moreover, treating bovine muscle cells with a cell-penetrating peptide derived from the syndecan-4 cytoplasmic region and knockdown analysis of syndecan-4 revealed that syndecan-4 is an important regulator of myoblast fusion and myogenetic transcription factors.

## Materials and Methods

### Antibodies

Immunofluorescence: Mouse anti-syndecan-4 (1:50 dilution, 5G9, SC-12766), goat anti-EEA1 (1:200 dilution) and goat anti-Lamin A/C (1:50 dilution, N-19) were from Santa Cruz Biotechnologies Inc. (Santa Cruz, CA, USA). Rabbit anti-GM130 (1:200 dilution) and rabbit anti-desmin (1:80 dilution) were from Abcam (Cambridge, UK). Mouse anti-HA antibody (1:100 dilution,), Alexa 488 goat anti-mouse, Alexa 546 goat anti-mouse, Alexa 488 goat anti-rabbit and Alexa 647-conjugated donkey anti-goat were from Invitrogen (Carlsbad, CA, USA). DyLight 549-conjugated mouse anti-rabbit were from Jackson ImmunoResearch Laboratories Inc. (West Grove, PA, USA). DAPI was from Molecular probes (Invitrogen, Paisley, UK). Immuno EM: Mouse anti-HA (12CA5, 1:250 dilution) was from Life science Roche (Penzberg, Germany), and rabbit anti-mouse (1:175 dilution) was from Cappel Research Reagents (ICN Biochemicals, Irvin, CA, USA).

### Bovine primary skeletal muscle cell isolation

Bovine primary skeletal muscle cells were isolated essentially as described [14]. The bovine primary skeletal muscle cells were obtained using fresh muscle samples from *Longissimus thoracis* (beef sirloin) collected at an industrial abattoir (Nortura AS, Rudshøgda, Norway). The cell cultures were isolated from animals of the same age, gender and breed. In brief, small muscle pieces (~ 1 g) were digested at 1 h/70 rpm shaking in 10 ml DMEM without FBS with 0.72 mg/ml collagenase. Cells were dissociated from the tissue by three treatments (25 min each) with 0.05% trypsin/EDTA. The harvested cells were pooled, and FBS (10%) was added after each treatment in order to inactivate trypsin. For removal of fast-adhering fibroblasts from the primary cell cultures, the cells were placed in uncoated cell flasks for 1 h at 37°C. This allowed the fibroblasts to adhere to the plastic. The non-adhering cells were then collected and further seeded onto 25 cm^2^ coated culture flasks until 50% confluence. The isolated cells were cultured, transferred into 75 cm^2^ coated culture flasks, and then stored in DMSO in liquid nitrogen until further use. All experiments were performed in 2^nd^ or 3^rd^ passage.

### Cell culture and treatment

Tissue culture coverslips (Menzel-Gläser, Braunschweig, Germany), 6- and 24-well plates (BD Falcon, Franklin Lakes, NJ, USA), or cell culture flasks (VWR, West Chester, PA, USA) were coated with 3 μl/cm^2^ Entactin-Collagen IV-Laminin (1 mg/ml, Millipore, Billerica, MA, USA). Subsequently the coated surface was washed twice with PBS. The primary cells were grown in Dulbecco’s modified Eagles’s medium (DMEM) with L-glutamine (2 mM), 2% FBS, 2% Ultroser G, P/S (10 000 units/ml), and Amphotericin B (250 μg/ml) until 70–80% confluence (usually 3 days). The cells were then washed with PBS and placed in differentiation medium (DMEM, 2% FBS, P/S, Amphotericin B, and 25 pmol insulin) to induce myogenesis.

### Transfection of cells

For protein knockdown experiments, confluent cells were transfected twice with a 24 h interval using Lipofectamine RNAiMax (Invitrogen) according to the protocols recommended by the manufacturers, using 33.75 μl lipofectamine and 750 pmol siRNA for each T-75 tissue culture flask. For syndecan-4 knockdown, the target sequences used were 5’-AGCCAAUACUUUUCCGGAGTT-3’ and 5’-CUCCGGAAAAGUAUUGGCUTT-3’. The duplexes were synthesized and annealed by Ambion (Life Technologies). Control cells were transfected with Stealth RNAi Negative Control Duplexes (Invitrogen). For transient transfection with plasmids, encoding syndecan-4-HA (SDC4-HA) and syndecan-GFP (SDC4-GFP), lipofectamine LTX (Invitrogen) was used according to the protocols recommended by the manufacturer. All transiently transfected cells were analysed 24–48 h after transfection.

### Peptide synthesis

Peptide arrays were synthesized as described [15]. In brief, peptide arrays were synthesized on cellulose paper using a Multipep automated peptide synthesizer (INTAVIS Bioanalytical Instruments AG, Koeln, Germany). Arginine coupled peptides were synthesized and purified to 80–95% purity (Genscript Corp, Piscataway, NJ, USA).

Arg_9_-Syn-4 cyt: R_9_-RMKKKDEGSYDLGKKPIYKKAPTNEFYA and Arg_9_ (control peptide): R_9._ Anti-syndecan-4 (sc-12766) blocking peptide: EGRYFSGALPDDEDVVGPGQESDDFELSG

### Peptide treatment of bovine muscle cells and toxicity analysis

Proliferating bovine muscle cells were grown to confluence, and treated with cell-penetrating Arg_9_ Syn-4 cyt (30 μM) for 24 h in differentiation media, and washed twice with PBS before analysis. Toxicity was measured as LDH release into the cell media and was performed according to protocol (cat. no. 11 644 793 001, Roche Applied Science, Mannheim, Germany). Incubation with 2% Triton X-100 for 2 h was used as positive control.

### Plasmid information

Syndecan-4-GFP: DNA coding green fluorescent protein (GFP) was fused into syndecan-4 in the extracellular domain after amino acid (aa) 84, and was kindly provided by Professor Kristian Prydz, University of Oslo, Norway. Syndecan-4-HA: The HA-tag was fused into the extracellular domain after aa 32, and was a gift from John Couchman, University of Copenhagen, Denmark [16].

### Antibody epitope mapping

Peptide membranes were blocked in 1% casein in TBST overnight at 4°C, incubated 2 h at room temperature with anti-syndecan-4 (sc-12766), washed three times 10 min in TBST, and incubated with a horseradish-peroxidase-conjugated secondary antibody for 1 h at RT before the washing steps were repeated. Chemiluminescence signals were developed by using Enhanced chemical luminescence Plus (RPN2132, GE HealthCare) and detected by Las 1000 (Fujifilm, Tokyo, Japan). For the peptide blocking experiment, anti-syndecan-4 was pre-incubated with the blocking peptide overnight at 4°C prior the immunoblotting procedure.

### RNA isolation and real-time PCR

Cell cultures treated as indicated in the figure legends were washed twice with PBS and purified by RNeasy mini kit including a DNase treatment (Qiagen, Hilden, Germany). cDNA was generated from ~200 ng mRNA using TaqMan Reverse Transcription Reagents (Invitrogen, Carlsbad, CA, USA) according to the manufacturer’s protocol. The cDNA was diluted four times before aliquots (in triplicates) were subjected to real-time PCR analysis using an ABI Prism 7700 Sequence Detection system (Applied Biosystem, UK). The real-time PCR reaction volume of 25 μl contained 4 μl template cDNA, 0.2 μM of each primer, 0.1 μM probe, 1.25 units Taq DNA polymerase (AmplitaqGold, Applied Biosystems, Carlsbad, CA, USA), 0.3 units uracil N-glycosylase (AmpErase UNG, Applied Biosystems), 0.2 mM dATP, dCTP, dGTP and 0.4 mM dUTP (Applied Biosystems), 5 mM MgCl2, and 1 x TBA buffer. The cycling profile was as follows: An initial decontamination step for 2 min at 50°C to allow optimal UNG enzymatic activity, followed by a denaturation step of 10 min at 95°C, followed by 40 repeats of 15 s denaturation at 95°C and 60 s synthesis at 60°C. A list of primers and probes used is provided in Table 1. Gene expression of the samples was normalized against β-actin, TATA and EF1, and ΔCt was calculated, according to the MIQE guidelines [17]. The results using TATA, and EF1 were similar, therefore only TATA was chosen for further analyses. PCR efficiency and melting point analysis were performed on all targets. Comparison of the relative gene expression between control and treated cells was derived by using the comparative Ct method. In short, values were generated by subtracting ΔCt values between two samples which gives a ΔΔCt value. The relative gene expression is then calculated by the formula 2^ΔΔCt^. The efficiency of each set of primers was always higher than 96%. The real-time PCR was performed in technical triplicates on at least three independent experiments seeded out in duplicates.

**Table 1:** List of primers and probes used for quantitative real-time PCR.

Primers and probes	Sequence	GeneBank acc. no
***Reference genes:***		
**β-Actin Forward**	CTGCGGCATTCACGAAACTA	**NM_173979**
**β-Actin Reverse**	GCACCGTGTTGGCGTAGAG	
**β-Actin Probe**	ATTCCATCATGAAGTGTGACGTCGACATCC	
**TATA Forward**	CGTTTTGCTGCTGTAATCATGAG	**NM_001075742**
**TATA Reverse**	CCATCTTCCCAGAACTGAATATCA	
**TATA Probe**	ATAAGAGAGCCCCGCACCACTGCA	
***Target genes:***		
**β-Actin Forward**	CTGCGGCATTCACGAAACTA	**NM_173979**
**β-Actin Reverse**	GCACCGTGTTGGCGTAGAG	
**β-Actin Probe**	ATTCCATCATGAAGTGTGACGTCGACATCC	
**MyoD Forward**	CCCAAAGATTGCGCTTAAGTG	**NM_001040478**
**MyoD Reverse**	AGTTCCTTCGCCTCTCCTACCT	
**MyoD Probe**	ACCACTCTCCTCCCAACAGCGCTTTAAA	
**Desmin Forward**	GCTGAAAGAAGAAGCGGAGAAC	**NM_001081575**
**Desmin Reverse**	GAGCTAGAGTGGCTGCATCCA	
**Desmin Probe**	ATTTGGCTGCCTTCCGAGCCG	
**Myogenin Forward**	CCCTACAGACGCCCACAATC	**EF636458**
**Myogenin Reverse**	AGCGACATCCTCCACTGTGAT	
**Myogenin Probe**	CACTCCCTCACCTCCATCGTGGACA	
**β1-Integrin Forward**	CCCTGATTGGTTGGTGGAAT	
**β1-Integrin Reverse**	TGCCACCAAGTTTCCCATCT	
**β1-Integrin Probe**	ACACGGCTGCTGGTGTTTTCCACA	**NM_174368**
**Syndecan-4 Forward**	GGGCAACCCAGTCCTGATCT	
**Syndecan-4 Reverse**	AGGTGATGTCTCTTCTGACACTGAA	
**Syndecan-4 Probe**	TCGGCATCTCCCGCTCTGATTACAG	**XM_584869**

### Immunocytochemistry and fluorescence microscopy

Cells were grown on coated coverslips (Assistent, Sondheim/Rhön, Germany), washed in PBS and fixed in either 2% PFA, 4% PFA (Reidel-de Haën, Seelze, Germany) or ice-cold ethanol for 15 min. The cells were washed three times in PBS, permeabilized using 0.1% Triton X-100 in PBS and incubated with 5% non-fat dry milk for 30 min before incubation with primary antibody for 1 h. Subsequent incubation with secondary antibodies was performed for 30 min before using Dako fluorescent mounting medium (Glostrup, Denmark). The cells were examined either by confocal microscopy (Leica TCS SP5, Mannheim, Germany) or by fluorescence microscopy analysis (apotome mode) (ZEISS Axio Observer Z1 microscope, Jena, Germany), and images were processed using Adobe Photoshop CS3. Brightness and contrast, if used, were adjusted manually across the entire image. The objective used by confocal microscopy (Leica) was HCX PL APO 1.25 oil, while the objective used with fluorescence microscopy was a LCI Plan-Neofluor 25x/ 0.8 1mm Korr M277 objective oil.

The fusion index (FI) (the number of cells with more than two nuclei) was calculated from two independent experiments and scored from at least four randomly chosen regions containing nuclei, and myotubes stained with anti-desmin. To quantify the number of myotube formation, at least six representative images per sample was scored.

### Immuno-electron microscopy (immuno-EM)

Confluent cells were transfected with the SDC4-HA construct, and induced to differentiate for three days in 10 cm dishes. Differentiated cells were fixed with a mixture of 4% PFA and 0.1% glutaraldehyde in in PBS and prepared for cryo-immuno EM as previously described [18]. Thawed cryosections were labelled using a mouse anti HA antibody (Roche Diagnostics GmbH), followed by rabbit anti mouse IgG (Cappel, ICN Biochemicals) and finally 15 nm protein A gold (purchased from G. Posthuma, Utrecht, the Netherlands).

### Statistical analysis

Statistical analysis was performed using the Mann-Whitney *u* test or a two-tailed, unpaired Student’s t-test, indicated in the figure legends. *P*-values < 0.05 were considered statistically significant and are indicated in each figure.

## Results

### Syndecan-4 internalizes from the plasma membrane and localizes to the nuclear membrane area during muscle differentiation

**Fig 1:**
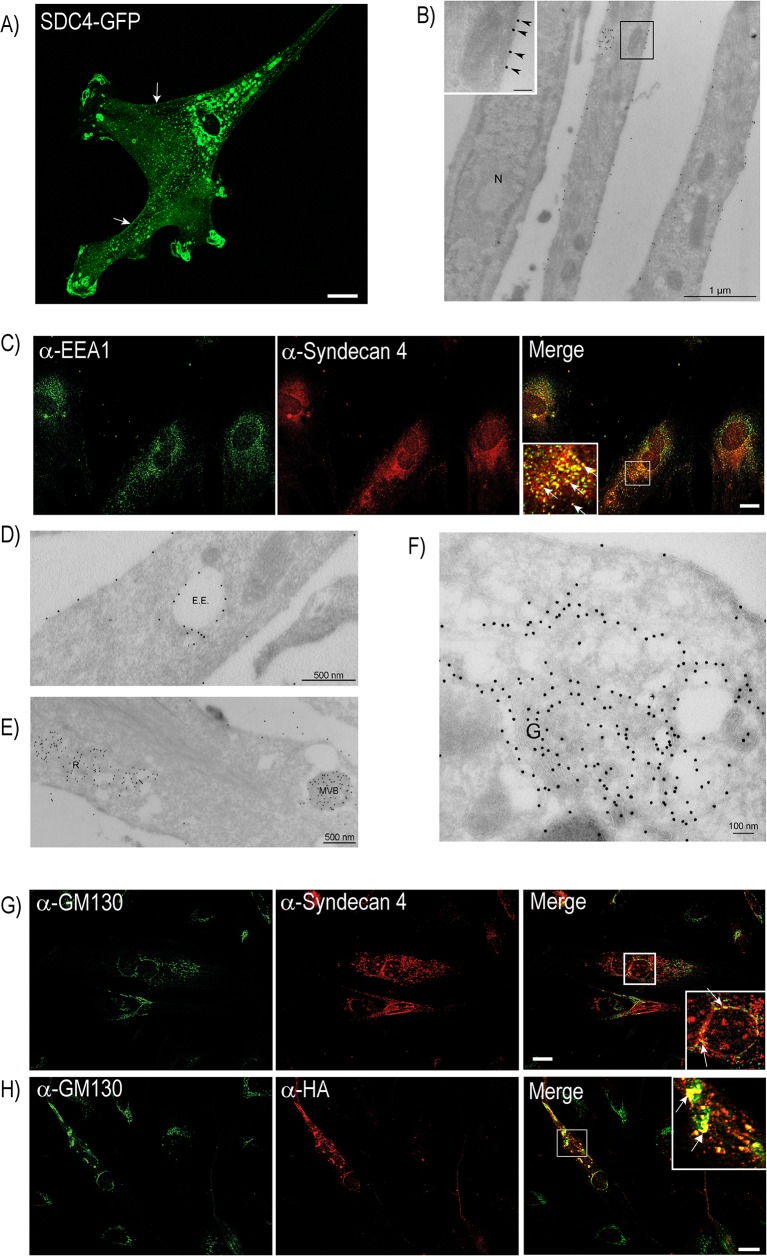
Syndecan-4 localization in bovine muscle cells. A) Muscle cells transiently transfected with Syndecan-4 (SDC4)-GFP were fixed with 4% PFA before fluorescence microscopy analysis. Scale bar: 20 μm. Arrows show plasma membrane staining. B) Muscle cells transiently transfected with SDC4-HA were induced to differentiation before immuno-EM preparation. Thawed cryosections were labelled using anti-HA antibody and 15 nm protein A gold. Arrow heads in insert (higher magnification of framed area) indicate syndecan-4 staining at the plasma membrane. N: Nucleus. Scale bar in insert: 100 nm. C) Differentiating cells, fixed with 4% PFA, were immunostained with mouse anti-syndecan-4 and goat-anti EEA1 (both diluted in PierceImmunostain Enhancer), followed by Alexa 546-conjugated goat anti-mouse (red) and Alexa 647-conjugated donkey anti-goat (green) before fluorescence microscopy analysis. The insert represents high magnification of the framed area. Arrows denote syndecan-4 and EEA1 co-localization. Scale bar: 20 μm. D-F) Thawed cryosections of SDC4-HA transfected cells induced to differentiation were labelled using an anti-HA antibody and 15 nm protein A gold. Localization of SDC4-HA to plasma membrane and compartments with morphology resembling D) early endosomes (E.E.), E) multi vesicular bodies (MVB) and compartments with a reticular morphology (R), and F) the Golgi apparatus (G.). G) Differentiating cells, fixed with ice-cold ethanol, were immunostained with mouse anti-syndecan-4 (diluted in Pierce Immunostain Enhancer) and rabbit anti-GM130, followed by Alexa 488-conjugated goat-anti rabbit (green) and Alexa 546-conjugated goat-anti mouse (red) before fluorescence microscopy analysis. The insert represents high magnification of the framed area. Arrows indicate co-localization of the Golgi marker GM130 and syndecan-4. Scale bar: 20 μm. H) Muscle cells transfected with SDC4-HA and induced to differentiated were fixed with ice-cold ethanol and immunostained with mouse anti-HA and rabbit anti-GM130, followed by Alexa 488-conjugated goat-anti rabbit (green) and Alexa 546-conjugated goat-anti mouse (red) before fluorescence microscopy analysis. The inserts represents high magnification of the boxed area. Arrow indicates co-localization of the Golgi marker and anti-HA. Scale bar: 20 μm.

To investigate the cellular localization of syndecan-4 GFP- or Hemagglutinin (HA)-tagged syndecan-4 were transiently overexpressed in cultured bovine muscle cells before confocal microscopy and immuno-EM analyses. Consistent with literature, GFP-tagged syndecan-4 localized to the plasma membrane and presumable focal adhesions (Fig 1A). Plasma membrane localization of HA-tagged syndecan-4 was confirmed by immuno-EM (Fig 1B). In addition, a vast portion of the protein was present in intracellular membranes. After double labelling for endogenous syndecan-4 and the early endosomal marker EEA1, a strong co-staining was observed (Fig 1C), indicating localization of syndecan-4 in the endocytic pathway. Closer inspection using immuno-EM showed that HA-tagged syndecan-4 localized to compartments with morphology resembling early endosomes (Fig 1D, labelled EE) and typical late endosomes with the morphology of multi vesicular bodies (Fig 1E, labelled MVB). In addition, immuno-EM showed strong labelling for HA-tagged syndecan-4 in membrane compartments with a more reticular like morphology (Fig 1E, labelled R). The nature of this reticulum is unknown, but occasionally it was observed in close association with the Golgi apparatus, which also labelled for HA-tagged syndecan-4 (Fig 1F, labelled G). To confirm this intracellular localization of syndecan-4 double staining of syndecan-4 and the Golgi marker GM130 was performed in differentiated muscle cells. Co-staining was observed between GM130 and both endogenous syndecan-4 (Fig 1G), and HA-tagged syndecan-4 (Fig 1H) in the perinuclear area of the cell.

**Fig 2:**
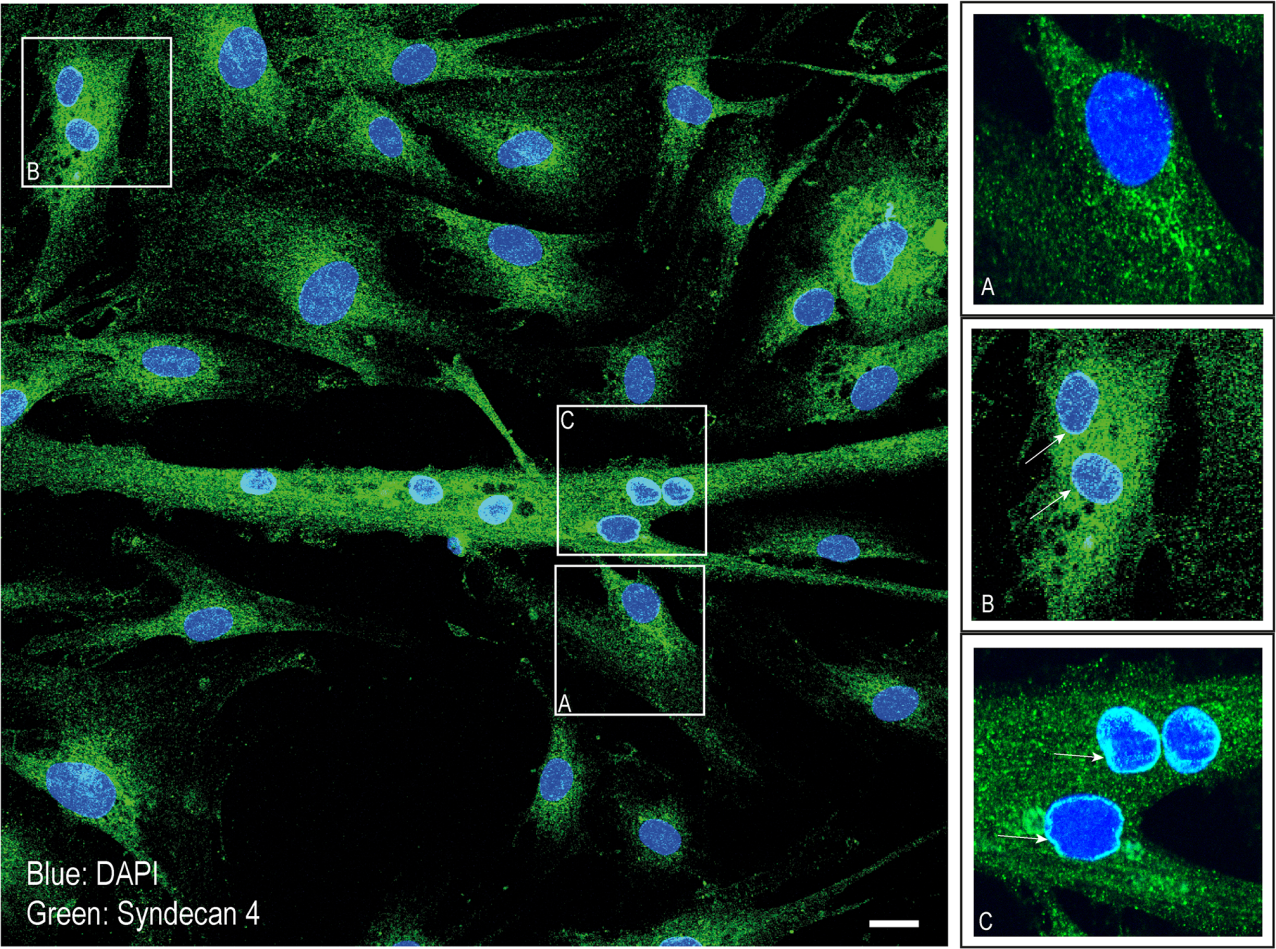
Localization of syndecan-4 to the perinuclear area during muscle cell differentiation. Myoblasts and myotubes induced to differentiate for three days were fixed with 4% PFA and immunostained with mouse anti-syndecan-4, followed by Alexa 488-conjugated goat anti-mouse (green) before confocal microscopy analysis. Nuclei were stained with DAPI (blue). The framed areas (A-C) are presented at high magnification in the right panels. Arrows denote nuclei with syndecan-4 localized to the nuclear membrane in early differentiated muscle cells (B) and in complex myotubes (C). Scale bar 20 μm.

Interestingly, closer inspection showed that endogenous syndecan-4 in some cells located not only to the Golgi, but also to areas close to the nucleus and along the nuclear membrane (Fig 2). The presence of syndecan-4 in the perinuclear area was apparent in cells at the initial myotube formation step (two myoblasts fused) (Fig 2, picture B). The staining along the nuclear membrane was strong and persistent upon differentiation into complex multinucleated myotubes (Fig 2, picture C). Only very weak perinuclear staining was observed in mononucleated cells (picture A versus picture B and C).

**Fig 3:**
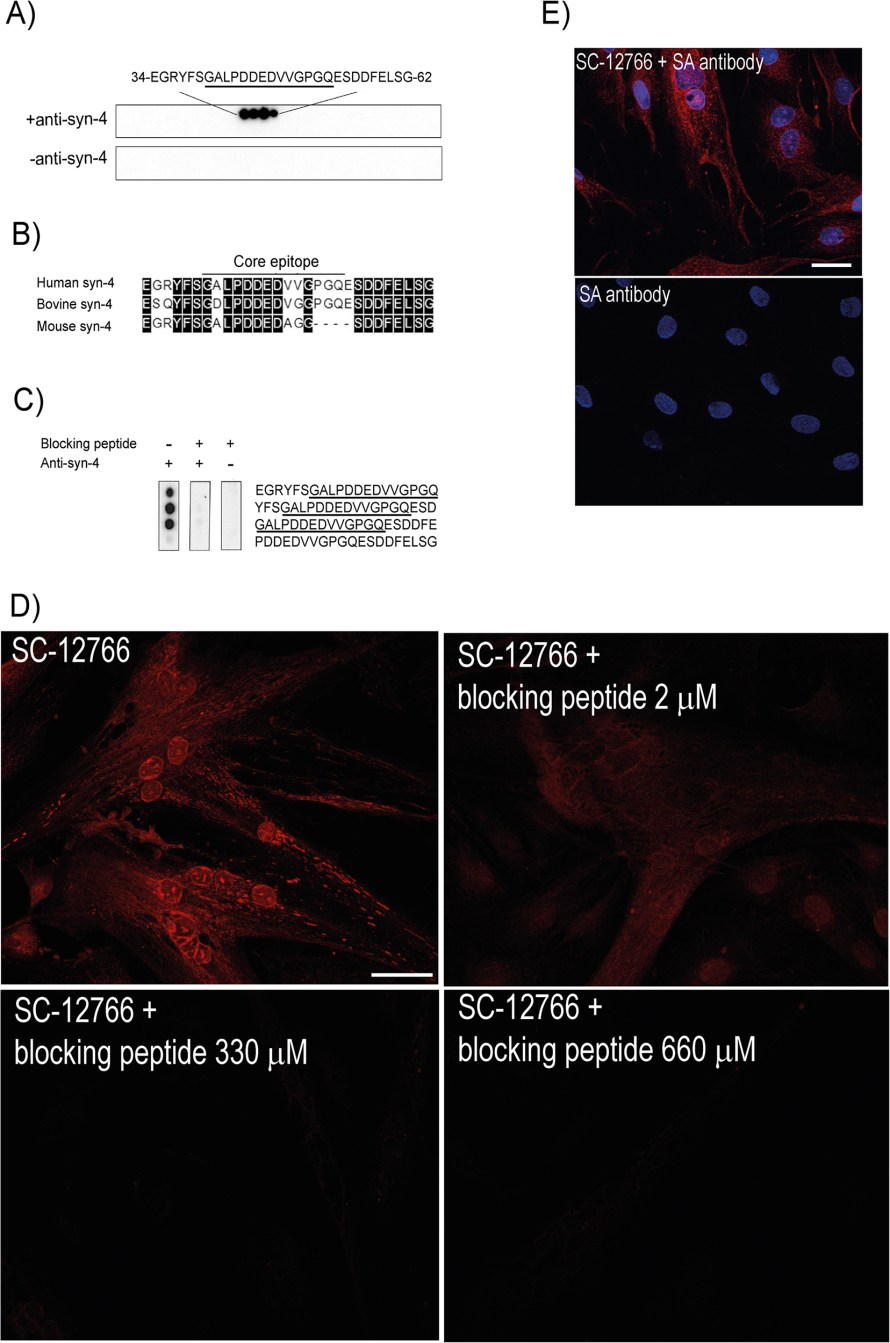
Epitope mapping of syndecan-4 antibody (sc-12766) and design of a specific blocking peptide. A) The antibody epitope was identified by overlaying an array of immobilized overlapping 20-mer human syndecan-4 peptides with anti-syndecan-4 (upper panel). Amino acids relevant for anti-syndecan-4 recognition are given. Underlined amino acids indicate the core epitope. Immunoblotting without anti-syndecan-4 was used as control (lower panel). B) Alignment of human vs. bovine and mouse syndecan-4 protein sequences. Black boxes indicate identical amino acids (DNA Star, Madison, Wisconsin). C) Amino acid sequences relevant for anti-syndecan-4 binding were synthesized on a membrane and overlaid with anti-syndecan-4 pre-incubated without (left panel) or with the blocking peptide (middle panel). Anti-syndecan-4 was omitted in the negative control (right panel). D) Staining of myoblasts and myotubes after three days in differentiating medium. Cells were fixed with ice-cold ethanol and immunostained with anti-syndecan 4 (sc-12766) alone (diluted in Pierce Immunostain Enhancer for increased antibody binding), or in combination with various concentrations of blocking peptide (2-330-660 μM), followed by Alexa 546-conjugated goat anti-mouse (red) before fluorescence microscopy analysis (ZEISS Axio Observer Z1 microscope). Scale bar: 50 μm. E) Control experiment with secondary antibody (SA) alone show little unspecific binding. Cells stained with anti-syndecan-4 followed by Alexa 546-conjugated goat anti-mouse (upper panel), or with secondary antibody (Alexa 546-conjugated goat anti-mouse) alone (lower panel) before fluorescence microscopy analysis (ZEISS Axio Observer Z1 microscope). Nuclei were stained with DAPI (blue). All images were captured using the same settings.

To exclude the possibility that the observation of endogenous syndecan-4 in the nuclear membrane area was a result of improper or false antibody binding, epitope mapping of the syndecan-4 antibody (sc-12766), which according to the manufacturer is raised against human syndecan-4, was performed (Fig 3A). Human syndecan-4 synthesized as 20-mer overlapping peptides on membranes was overlaid with anti-syndecan-4. Anti-syndecan-4 strongly recognized amino-acids 34–62 in human syndecan-4 (core epitope underlined) (Fig 3A). Incubation without anti-syndecan-4 was used as a negative control. Alignment of human, bovine and mouse syndecan-4 protein sequences revealed that the core epitope was almost identical between human and bovine syndecan-4, differing with only two amino acids (Fig 3B). Identification of the epitope allowed us to design a soluble blocking peptide able to neutralize the syndecan-4 antibody. As shown in Fig 3C anti-syndecan-4 was not able to recognize syndecan-4 when the antibody was pre-incubated with an excess of the blocking peptide (middle panel). Anti-syndecan-4 without the blocking peptide and incubation without primary antibody was used as positive and negative controls (left and right panels, respectively). Differentiated bovine muscle cells were stained with anti-syndecan-4 antibodies that had been pre-incubated with or without the designed blocking peptide. Importantly, using the same microscopy settings, fluorescence microscopy analysis showed no staining for endogenous syndecan-4 when anti-syndecan-4 was pre-incubated with various concentrations of the blocking peptide (Fig 3D). Neither was any staining detected when the primary antibody was omitted. (Fig 3E). Altogether, these results strongly suggest that the signal from the syndecan-4 antibody in muscle cells to be specific.

**Fig 4:**
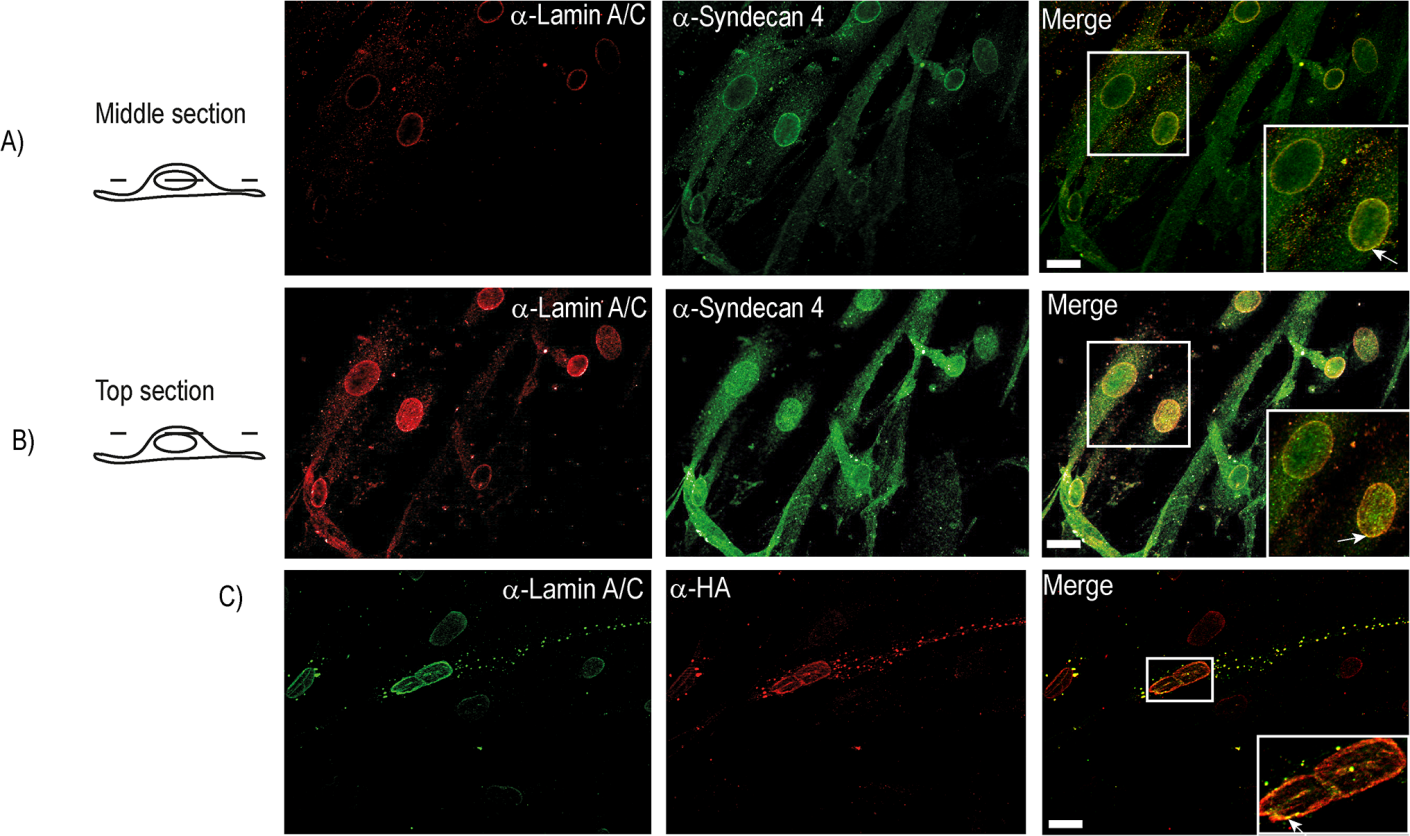
Syndecan-4 co-localizes with the nuclear protein Lamin A/C. A-B) Myoblasts and myotubes induced to differentiate for three days were fixed with 4% PFA and immunostained with mouse anti-syndecan-4 and goat-anti Lamin A/C, followed by Alexa 546-conjugated goat anti-mouse (green) and Alexa 647-conjugated donkey anti goat (red) before confocal fluorescence microscopy analysis (ZEISS Axio Observer Z1 microscope). The micrographs show the middle section (A) and the top section (B) of the nuclei. The inserts in the right panel show higher magnification of the framed areas. Arrows denote areas within the nuclear membrane which appear positive for both syndecan-4 and Lamin A/C. C) Muscle cells transiently transfected with SDC4-HA were immunostained with mouse anti-HA and goat Lamin A/C, followed by Alexa 647-conjugated donkey-anti goat (green) and Alexa 546-conjugated goat-anti mouse (red) before fluorescence microscopy analysis. Arrow indicates co-localization of Lamin A/C and anti-HA in the nuclear membrane. The inserts in the right panel shows higher magnification of the framed area. Scale bars: 20 μm.

**Fig 5:**
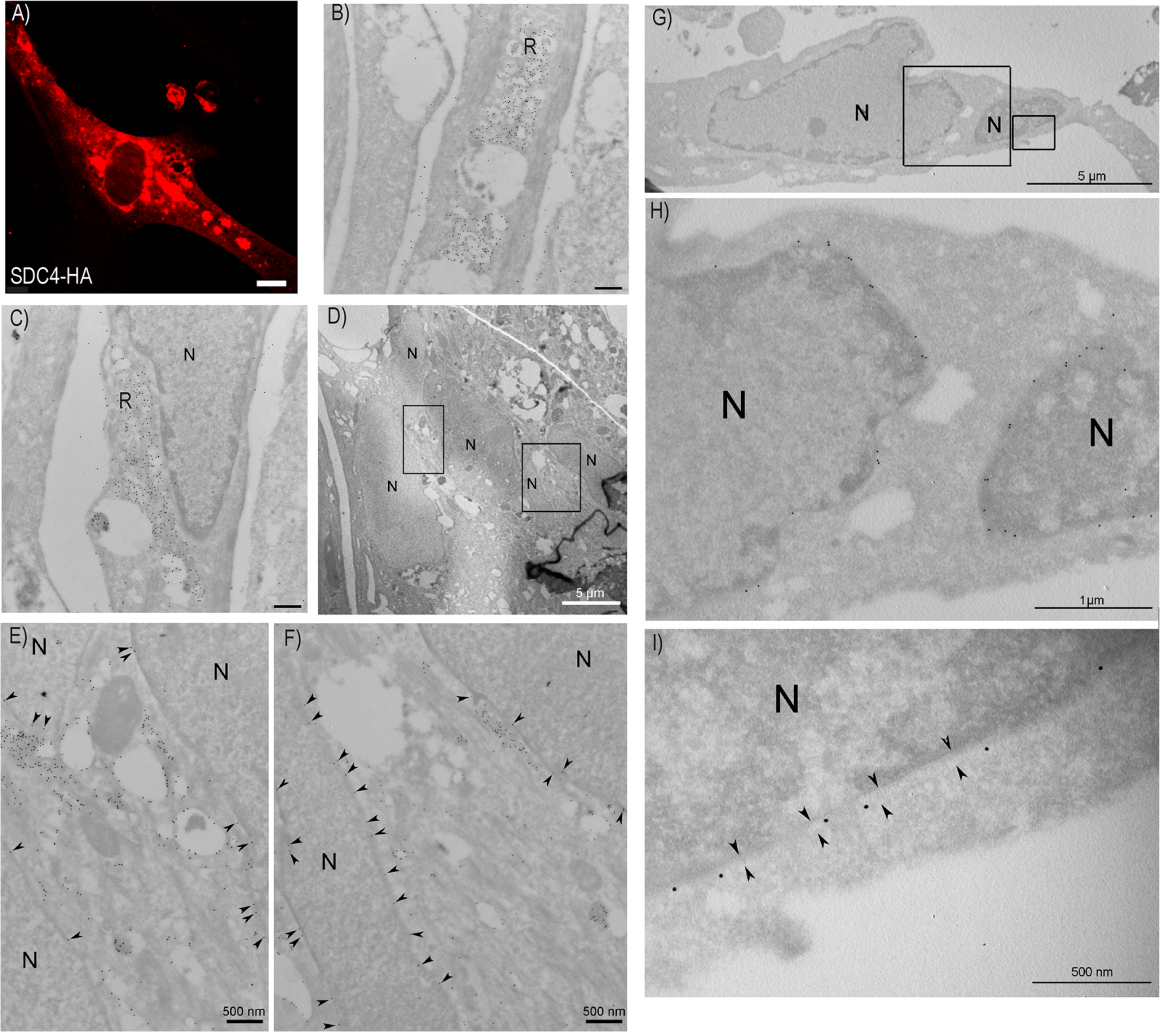
Syndecan-4 localizes to the nuclear envelope during muscle cell differentiation. A) Muscle cells were transiently transfected with SDC4-HA, fixed with 4% PFA and immunostained with mouse anti-HA, followed by Alexa 546-conjugated goat anti-mouse (red) before confocal microscopy analysis. Labelling was found throughout the cytosol, as well as perinuclear along the nuclear envelope. Scale bar: 10 μm. B-I) Immuno-EM localization of syndecan-4 HA in reticular compartments (B-C) and the nuclear envelope (D-I). Muscle cells transiently transfected cells with SDC4-HA were induced to differentiation before preparation for immuno EM. Thawed cryosections were labelled using anti-HA antibody and 15 nm protein A gold. B-C) Transfected cells showed strong labelling in the membrane of reticular compartments (R) which, as shown in C), often was localized close to the cell nucleus (N). Scale bars: 500 nm. D) Overview of a differentiated cell showing multiple nuclear profiles. Scale bar as indicated. E-F) Higher magnifications of framed areas in D. Arrow heads indicate syndecan-4 labelling localized to the nuclear envelope. N: Nucleus. Scale bars as indicated. G) Overview of parts of a multinucleated cell. H-I) Higher magnifications of the framed areas demonstrate labelling associated with the nuclear envelope. Arrow heads in I indicate the inner and outer nuclear membrane. N: Nucleus. Scale bars as indicated.

In order to study the perinuclear localization of syndecan-4 during differentiation in more details, double staining of endogenous syndecan-4 with the nuclear membrane protein lamin A/C was performed. Strong co-localization of syndecan-4 and lamin A/C was observed in both the middle section and the top section of the nucleus in differentiated cells (Fig 4A and 4B). Co-localization of syndecan-4 with lamin A/C in the perinuclear region was also observed for transiently express HA-tagged syndecan-4 (Figs 4C and 5A). Localization of HA-tagged syndecan-4 in the perinuclear area was also analysed by immuno-EM. Consistent with Fig 1, HA-syndecan-4 localized in compartments with a reticular morphology (Fig 5B), which in many cells localized close to the nucleus (Fig 5C). Interestingly, close inspection of the nuclear area showed that HA-syndecan-4 located within the nuclear envelope in multinucleated cells (Fig 5D–5I, E and F are enlargements of D, whereas H and I are enlargements of G). Noteworthy, no significant labelling was observed within the nucleoplasma, indicating that localization of syndecan-4 was restricted to the nuclear envelope.

**Fig 6:**
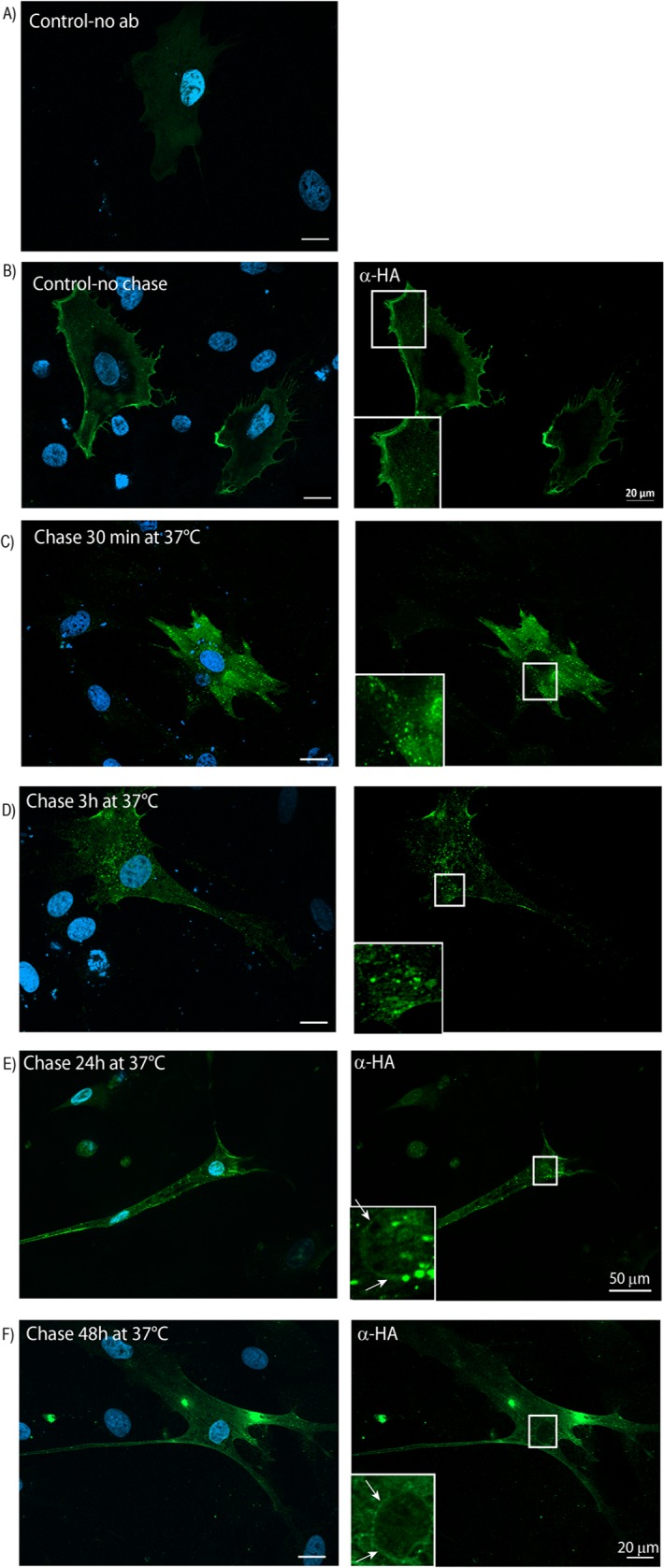
Syndecan-4 is internalized from the plasma membrane to the nuclear membrane. Muscle cells transiently transfected with SDC4-HA were either left untreated (A) or incubated with mouse anti-HA antibody for 30 min on ice to allow binding of antibody to SDC4-HA at the plasma membrane. The cells were then washed with ice-cold PBS and either fixed immediately (B), or chased for 30 min (C), 3 h (D), 24 h (E) and 48 h (F) at 37°C in differentiation media before fixation. After permeabilization, localization of anti-HA was detected using an Alexa 488-conjugated anti-mouse antibody (green). Nuclei were stained with DAPI (blue) (A-F). The right panels show only the anti-HA staining. This allows a better visualization of internalized anti-HA localized to intracellular compartments and the nuclear membrane. The inserts (framed areas at high magnification) show staining for HA in the nuclear membrane (denoted by arrows). Scale bars as indicated.

To investigate the dynamics of syndecan-4 during differentiation, and if the perinuclear syndecan-4 represented newly synthesized syndecan-4 or was syndecan-4 internalized from the plasma membrane during differentiation, muscle cells were transiently transfected with HA-syndecan-4 and incubated on ice with an HA-antibody. The cells were then washed to remove unbound antibody, and either fixed immediately or chased for 30 min, 3h, 24 h or 48 h at 37°C in differentiation media before fixation. Transfected cells without any antibody incubation were used as a negative control (Fig 6A). When staining for the HA-antibody, cells incubated on ice displayed clear plasma membrane labelling (Fig 6B), whereas cells chased for 30 min, 3h. 24 and 48h at 37°C showed labelling in intracellular compartments, an additionally labelling in perinuclear areas was observed at longer chasing time points (Fig 6E and 6F, enlargements of perinuclear regions are shown in the left corner of E and F).

### Syndecan-4 function as a negative regulator of muscle differentiation

**Fig 7:**
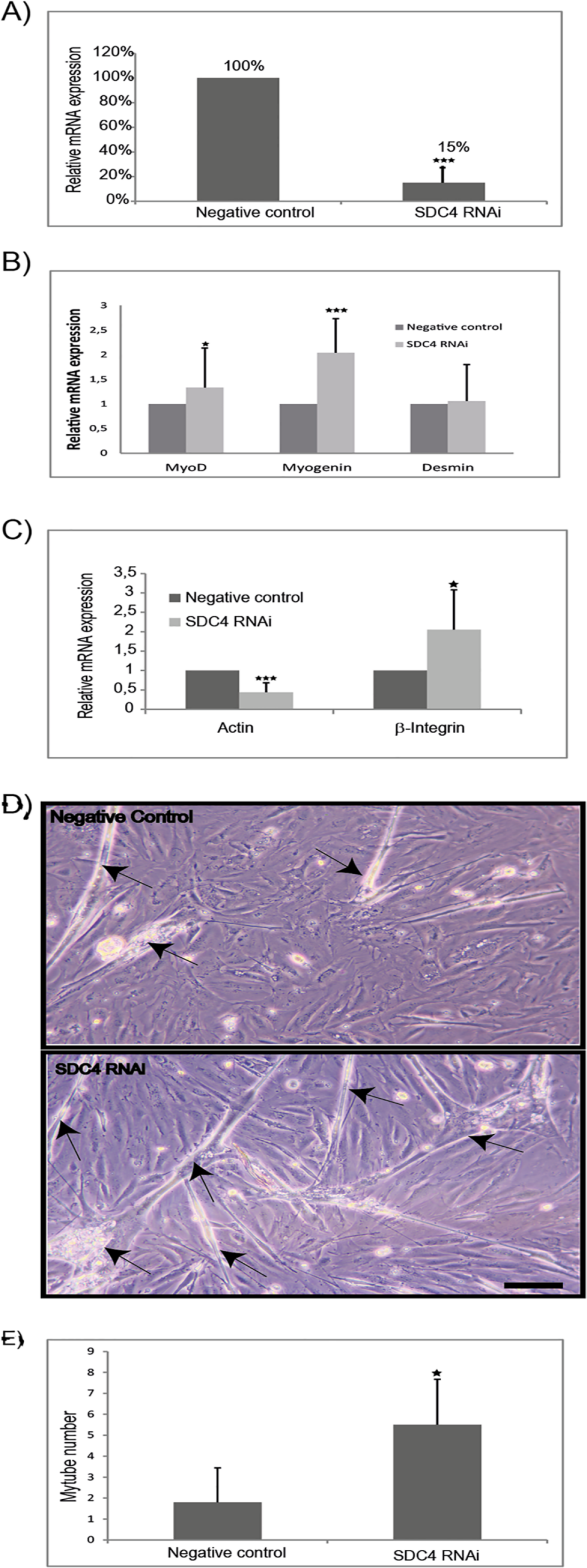
Syndecan-4 regulates myogenic transcription factors during muscle cell differentiation. A) Confluent cells were transfected with siRNA for negative silencer (negative control) or syndecan-4 (SDC4 RNAi), and induced to differentiate for three days. Bars show the relative mRNA expression of syndecan-4 in SDC4 RNAi treated cells compared to negative control cells measured after three days in differentiation media. B) Muscle cells were treated as described in A. Bars show the relative mRNA expression of MRFs in SDC4 RNAi treated cells compared to negative control cells. C) Muscle cells were treated as described in A. Bars show the relative mRNA expression of actin and Integrin β1 in SDC4 RNAi treated cells compared to negative control cells. The data in A-C is presented as the average mean of at least three independent experiments performed in technical triplicates, ± SD. Asterisks denote significant differences (*p<0.05, ***p<0.001). D) Cells treated with RNAi as described in A, showed increased myotube formation compared to negative control cells. Arrows indicate myotubes formation. Scale bar 5 μm. E) Quantification of myotube numbers. A total of at least 6 representative images per sample were scored for myotube number formed. Asterisk denote significant differences (*p<0.05).

Syndecan-4 has been suggested to play an important role in early stages of myogenesis, and adult satellite cells from syndecan-4^-/-^ mice are unable to reconstitute damaged muscle [8]. To further investigate the importance of syndecan-4 in differentiation of bovine muscle cells, RNAi was used to down-regulate the syndecan-4 mRNA expression. The siRNA transfected cells were induced to differentiate for three days before various analyses. The knock down of syndecan-4 reduced the mRNA expression level to 15% compared to expression in control cells treated with negative silencer siRNA (Fig 7A). By real-time PCR of established myogenic regulatory transcription factors and myogenic markers we observed significant differences in syndecan-4 knockdown cells compared to negative control cells (Fig 7B and 7C). The mRNA level of the early differentiation markers MyoD and myogenin were increased (Fig 7B). Inhibition of syndecan-4 expression did not affect the mRNA expression level of the filamentous protein desmin. Interestingly we observed a significant increase in the mRNA expression of the cell surface integrin β1, which is known to associate with syndecan-4 in focal adhesions at the plasma membrane [3]. In contrast, the expression level of actin mRNA showed a significant decrease (Fig 7C). Importantly, visual inspection by light microscopy indicated that syndecan-4 knockdown positively affected the formation of myotubes (Fig 7D), and quantification showed that the number of observed myotubes increased in syndecan-4 knock down cells (Fig 7E).

### The cytoplasmic domain of syndecan-4 is involved in *myoblast fusion*

**Fig 8:**
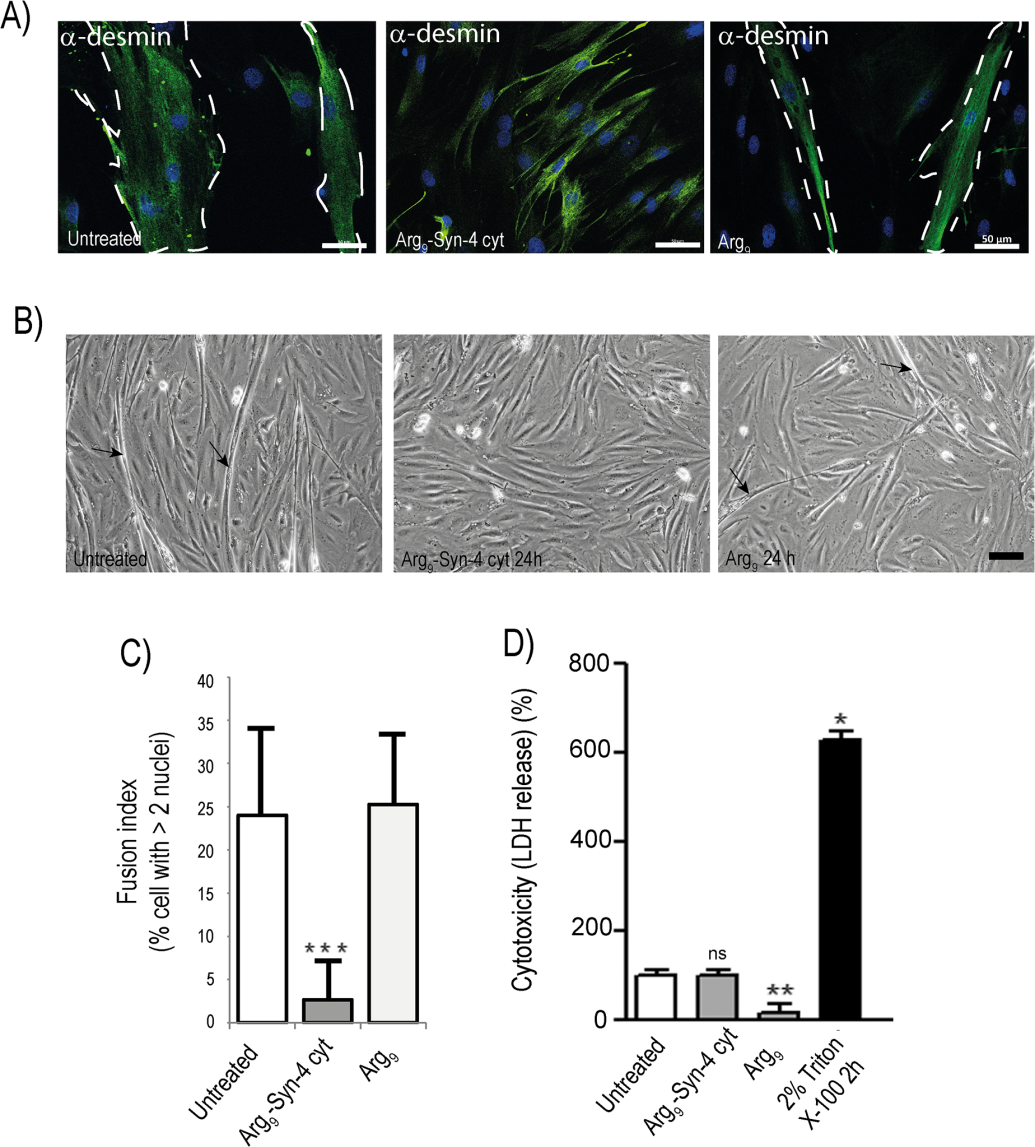
The cytoplasmic domain of syndecan-4 is involved in myoblast fusion. A-B) Confluent muscle cells incubated without (left panel), or with either the cell-penetrating peptide Arg_9_-Syn-4 cyt (middle panel) or the cell-penetrating Arg_9_ control peptide (right panel) for 24 h in differentiation media. A) Fluorescence microscopy analysis of cells stained with anti-desmin (green) and DAPI (nuclei, blue) after fixation with 4% PFA. Scale bars: 50 μm. Dashed area show myotubes in untreated cells (left) and in cells treated with control peptide (right panel), but no myotubes are observed in cells treated with Arg_9_-Syn-4 cyt (middle panel). B) Light microscopy analysis of muscle cells treated as in A. Arrows indicate myotubes in left and right panels (untreated cells and cell treated with the cell-penetrating Arg_9_ control peptide). Note that myotubes cultured in dishes will have a mixture of morphological characteristics, both branched and unbranched. It should be emphasized that the important observation in this experiment is the complete absence of myotubes in Arg_9_-Syn-4 cyt treated cells (Fig 8A and 8B, middle panels). Scale bar: 5 μm. C) The fusion index (FI) (the number of cells with more than 2 nuclei) was calculated based on scoring at least four randomly chosen regions with nuclei and myotubes stained as in A in three independent experiments. The FI was calculated as the percentage of total nuclei incorporated into myotubes. Asterisk denote significant differences between untreated and Arg_9_-Syn-4 cyt treated cells (**p<0.01, n>50 cells). Arg_9_ was used as a control peptide. D) Media from untreated muscle cells, muscle cells treated with Arg_9_-Syn-4 cyt and with Arg_9_ control peptide for 24 hours were subjected to LDH release analysis. Incubation with 2% Triton X-100 for 2 h was used as a positive control for the assay. Differences in release was tested by Mann Whitney U test (*p<0.05, n = 3–6). Error bars indicate SEM.

To study the impact of the syndecan-4 cytoplasmic domain on muscle differentiation, a cell-penetrating peptide containing the cytoplasmic region of syndecan-4 (Arg_9_-Syn-4 cyt) was used [15]. This peptide, made cell-penetrating through an N-terminal arginine-tag [19], is reported to compete with endogenous syndecan-4 for interaction with syndecan-4 binding proteins, resulting in a defect syndecan-4 signalling [15]. Confluent muscle cells, at a stage when no myotubes yet were formed, were incubated with the Arg_9_-Syn-4cyt peptide in differentiation media for 24 h. Preferably, we would have treated the muscle cells with the Arg_9_-Syn-4 cyt peptide for three days (similar timeline to the other experiments performed in this study), but treatment for longer periods leads to degradation of peptides [19]. Our data demonstrated that the muscle cells were already committed to differentiation after 24 h treatment, as indicated by expression of the filamentous protein desmin (a differentiation marker in bovine muscle cells) (Fig 8A, left panel). Surprisingly, the Arg_9_-Syn-4 cyt peptide inhibited myotube formation (Fig 8A and 8B, middle panels). The peptide-treated cells aligned to each other; ready for fusion, but few myotubes were formed. An Arg_9_ peptide (without the syndecan-4 derived sequence) was used as a negative control to exclude non-specific effects from the cell-penetration sequence (Fig 8A and 8B, right panels). No effect on myotube fusion was observed in this control experiment. Conclusively, the fusion index (i.e. % of cells with more than 2 nuclei) was significantly lower in cells treated with the Arg_9_Syn-4 cyt peptide compared to untreated cells or cell treated with the cell-penetrating Arg_9_ control peptide (Fig 8C). The peptides did not induce any LDH release into the media (Fig 8D), suggesting the observed effect of the Arg_9_-Syn-4 cyt peptide was specific, and not due to toxicity. Conclusively, our data indicate that the cytoplasmic domain of syndecan-4 inhibits the myoblast fusion process.

**Fig 9:**
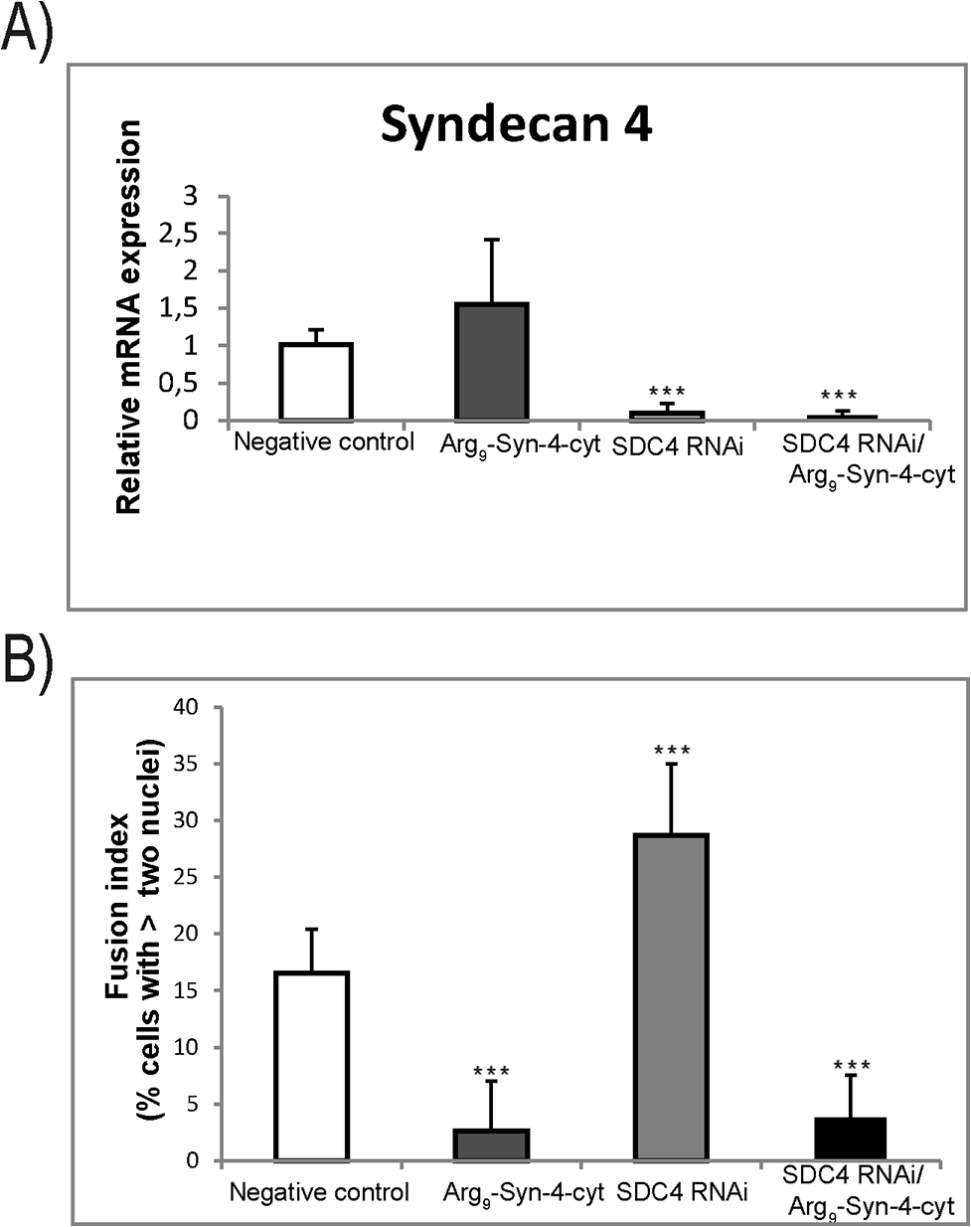
Syndecan-4 is a negative regulator of myogenesis. Confluent cells grown in differentiation media were either I) incubated for 24 h, II) treated with the cell-penetrating peptide Arg_9_-Syn-4 cyt for 24 h, III) transfected with SDC4 RNAi and incubated 24 h, and IV) transfected with SDC4 RNAi and treated with the cell-penetrating peptide Arg_9_-Syn-4 cyt for 24 h. A) Bars show the relative mRNA expression of syndecan-4 compared to negative control cells measured after 24h in differentiation media. The data in A is presented as the average mean of at least two independent experiments seeded out in duplicates, performed in technical triplicates, ± SD. Asterisks denote significant differences (***p<0.001). B) The fusion index (FI) (the number of cells with more than 2 nuclei) was calculated based on scoring at least six randomly chosen regions with nuclei and myotubes stained as in Fig 8A, in two independent experiments. Asterisk denote significant differences between negative control cells, compared to SDC4 RNAi and SDC4 RNAi + Arg_9_Syn-4 cyt treated cells (***p<0.001, n>100 cells).

In a final experiment, the siRNA experiment was combined with the Arg_9_-Syn-4 cyt peptide. The Arg_9_-Syn-4 cyt peptide neither affected the syndecan-4 mRNA expression in cells treated with the peptide nor the knock down of syndecan-4 by siRNA (Fig 9A). However, surprisingly the Arg_9_-Syn4 cyt peptide reduced the myotube formation in the siRNA treated cells, suggesting the peptide to overrule the positive effect of syndecan-4 knock-down (Fig 9B). Altogether, these results demonstrate that syndecan-4 has important regulatory functions in muscle differentiation of bovine muscle cells *in vitro* and that syndecan-4 through its cytoplasmic part is a negative regulator of myogenesis.

## Discussion

### Cellular localization of syndecan-4 during differentiation in bovine muscle cells

The proteoglycan syndecan-4 is a cell surface HSPG that functions as receptor for several types of ligands and is thus important for the interplay between extracellular matrix and the cell interior [5]. A number of reports, however, suggest that HSPGs also have roles intracellularly in processes such as sorting of proteins for secretion, nuclear processes and storage of granule constituents [4, 20–22]. In this study we demonstrate that syndecan-4, in addition to being localized to the plasma membrane, also localizes in endocytic compartments such as early endosomes and multivesicular bodies, indicating internalization and trafficking along the endosomal/lysosomal degradation route [23, 24] during muscle cell differentiation. Since recent findings demonstrate that the syndecan-4/syntenin complex is essential for exosome biosynthesis [25], and multivesicular bodies give rise to exosomes (reviewed in [26]), this localization could also be relevant to inter-cellular signalling through the release of exosomes. Data from mice muscle cells show that exosome-like vesicles from myotubes induce growth arrest and commit cells to differentiation [27]. Most interestingly, our experiments show that syndecan-4 is internalized from the plasma membrane during muscle cell differentiation, and that internalized syndecan-4 during this process localizes to the nuclear envelope. A clear shift in localization was observed, from cell surface and cytoplasmic localization in proliferating cells, to the nuclear area in the differentiated cells. Some of the syndecan-4 observed in the perinuclear region does probably represent newly synthesised syndecan-4. However, the localization of internalized anti-HA antibodies in intracellular compartments and perinuclear areas (Fig 6C–6F) clearly demonstrates a retrograde transport of HA-syndecan-4, probably all the way back to the nuclear envelope. It is likely that syndecan-4 in one or more of the endocytic compartments, escapes the degradation route, and is alternatively routed to the nuclear membrane area. This shift in localization is apparently important at early stages of muscle cell differentiation, as a weak staining was observed already in some mononucleated cells committed to differentiation and cells with only two nuclei show strong perinuclear staining. This fascinating topic has so far not been the subject of detailed investigations, although syndecan-4 has been observed in nuclear or perinuclear areas in breast carcinoma [28]. Since neither confocal microscopy nor immuno-EM showed any significant labelling for syndecan-4 (endogenous or HA-syndecan-4) within the nucleoplasma, localization of syndecan-4 seems to be restricted to the nuclear envelope. However, due to the size of the labelling reagents used for immuno-EM (primary anti-HA antibody + secondary anti-mouse antibody + protein A coated 15 nm gold) we were not able to determine whether syndecan-4 located specifically to either the outer or the inner membrane of the envelope. Moreover, since all the antibodies used in this study recognize the extracellular part of syndecan-4 (endogenous or HA-syndecan-4), we were not able to decide whether syndecan-4 present in the nuclear membrane represents full-length or processed syndecan-4. Thus we cannot exclude the possibility that the intracellular domain of syndecan-4 is present within the nucleoplasma.

The presence of PGs in the nucleus is still somewhat debated, and early studies demonstrating nuclear PG have been claimed to be results of contamination [22]. This view is now changing as new methods are emerging, especially immunocytochemistry techniques that provide possibilities for a more precise determination of intracellular localization of HSPGs [4]. Intact syndecan-1 contain a nuclear localization sequence RMKKK in the cytoplasmic domain, and this sequence is necessary for translocation of the full-length syndecan-1 to the nucleus in mesenchymal tumour cells [29]. By bioinformatics syndecan-4 was found to contain an RMKKK motif identical to syndecan-1. The RMKKK motif is also the minimal motif important for efficient raft dependent endocytosis of syndecan-1 [30], suggesting a connection between endocytosis and nuclear translocation. The importance of endocytosis for cell-cell fusion of myoblasts into multinucleated myotubes has recently been demonstrated [31]. Baron *et al* demonstrated that endocytosis is indeed required for myotube formation, and they suggested that endocytosis contributes to the membrane fusion itself. Several growth factor receptors locate to the nucleus [32]. The epidermal growth factor receptor (EGFR) is suggested to be transported through a retrograde route from the cell surface to the nucleus via the endoplasmic reticulum (ER) [32]. Also CD44 (a cell-surface glycoprotein involved in cell–cell interactions, cell adhesion and migration) is internalized and upon endosomal sorting, it is imported to the nucleus through the nuclear pore complex [33].

### Muscle cell differentiation is tightly regulated by syndecan-4

Both the observed internalization of syndecan-4 and our knock down data support the notion the plasma membrane localized syndecan-4 may function as a negative regulator of muscle differentiation. A potential negative role for the syndecan-4 extracellular domain in muscle differentiation is that it through its binding of various ligands participates in signalling to the cell interior [1]. Syndecans are responsible for recruiting soluble growth factors such as FGF to the cell membrane and act as co-receptors for FGFR. The growth factor FGF2 is a strong inhibitor of skeletal muscle differentiation, and it diminishes the expression of myogenin [34]. Due to reduced amount of FGF2 bound and presented to the FGF receptor upon syndecan-4 internalization or knockdown, FGF2-FGFR signalling will be reduced, leading to decline of cell proliferation, and increased muscle differentiation. In support of this we found that knock down of syndecan-4 caused an increase in myogenin expression and increased myotube formation. These findings are in line with results obtained in turkey primary muscle cells after syndecan-4 knockdown, where they observed increased MRF expression [35] and muscle differentiation [36]. Likewise, inhibiting syndecan-3 expression in C2C12 cells resulted in increased expression of myogenin and increased myoblast fusion [37]. We also found that the expression of actin was decreased in syndecan-4 knock down cells and the β1-integrin expression was up-regulated. β1-integrin is constitutively expressed in skeletal muscle, and is shown to mediate muscle differentiation [38]. Syndecan-4 is an important component of focal adhesions (FA), and for integrin-mediated focal adhesion formation [5]. In normal cells, endocytosis and recycling of integrins modulate FA turnover and proper cell migration [39]. The up-regulation of β1-integrin observed herein suggests that regulation of expression and the steady-state of syndecan-4 and β1-integrin are somehow connected in muscle cells. How syndecan-4 and integrin interacts is still not known, but Song et al showed that N-glycosylated chains on syndecan-4 are critical for the syndecan-4 and integrin interaction, and that deletion of these chains reduced FAK activity in turkey muscle cells [40].

### The syndecan-4 cytoplasmic domain is important for myoblast fusion during muscle differentiation

The above discussed data point out important roles of the syndecan-4 extracellular domain, this does however, not exclude that also the cytoplasmic tail of syndecan-4 participate in regulation of myogenesis. Cornelison *et al* suggested that syndecan-4 activities reside in the syndecan-4 protein rather than the attached GAG chains, since rescue experiments with exogenous soluble heparin (closely related to heparin sulfhate; hence mimicking the function of GAG chains) showed minimal improvement on muscle differentiation [8]. When we treated the bovine muscle cells with a cell-penetrating Arg_9_-Syn-4 cyt peptide (containing the same sequence as the syndecan-4 cytoplasmic tail), we observed a dramatic effect on myoblast fusion. The cells were committed to differentiation, expressed desmin and were aligned nicely to each other, but no myotubes were formed. Since our findings indicate that internalization and transport of syndecan-4 to the nuclear envelope is essential for muscle cell differentiation, the effect of the Arg_9_-Syn-4 cyt peptide could possibly be inhibition of an interaction between endogenous syndecan-4 and an adaptor protein involved in its internalization and/or endosomal sorting. Dishevelled, a component of the Wnt-signalling pathway, is suggested to function as a bridge both between syndecan-4 and adaptor protein-2, and between syndecan-4 and ubiquitin ligases, and as such regulate syndecan-4 endocytosis and degradation respectively [23]. The intracellular protein syntenin which also binds syndecan-4, promotes recycling from endosomal compartments to the plasma membrane [41], suggesting that internalization and recycling versus degradative sorting of syndecan-4 within endosomal compartments is tightly regulated. However, treatment with the Arg_9_-Syn-4 cyt peptide also inhibited the increased myoblast fusion otherwise observed upon syndecan-4 knockdown. This finding indicates that the cytoplasmic domain of syndecan-4 itself, possibly through its unique V-region inhibits myoblast fusion *in vitro*. The V-region of syndecan-4 binds to the membrane lipid phosphatidylinositol 4,5 bisphosphate (PtdIns4,5P_2_), and this complex binds and activates PKCα [5, 12]. The direct binding of PKCα to syndecan-4 increases PKCα localization in focal adhesions [42]. Moreover, focal adhesion formation is initiated through syndecan-4 activation of either PKCα[40] or FAK [43]. Interestingly, FAK activity is required for myoblast fusion [44, 45]. Thus, inhibition of myoblast fusion with the Arg_9_-Syn-4 cyt peptide might arise from inhibited syndecan-4 FAK activation. However, the increased myoblast fusion observed upon syndecan-4 knock down shows that myogenesis does not depend on syndecan-4 mediated FAK activation. Other possibilities are thus that the cytoplasmic domain of syndecan-4 either interacts with and activates an inhibitor of myoblast fusion, or alternatively, inhibits an activator protein needed for myoblast fusion. Both processes will inhibit myoblast fusion and explain why syndecan-4 knockdown increased myoblast fusion. If any of such interactions occur at the plasma membrane, it will also explain how the observed internalization of syndecan-4 regulates myotube formation.

**Fig 10:**
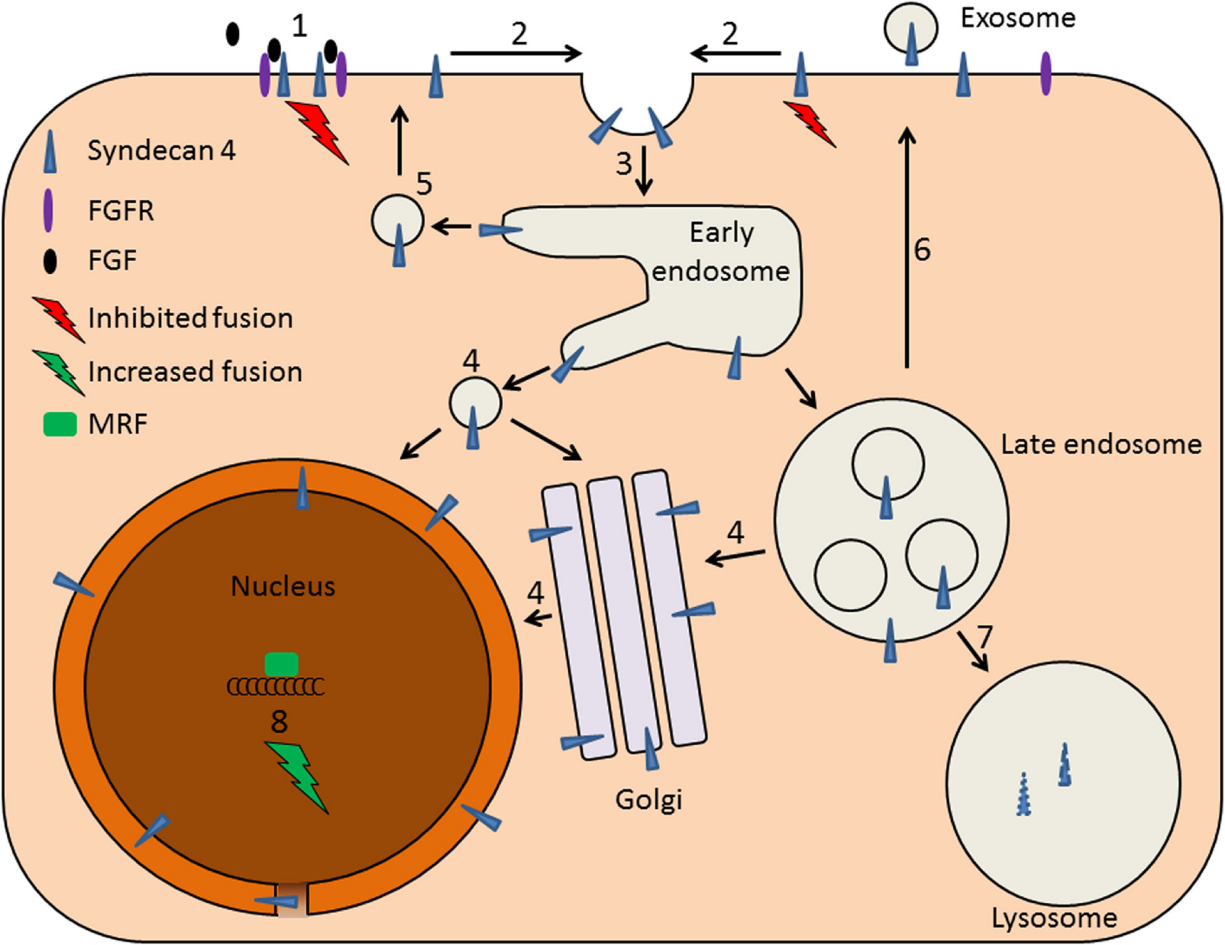
Tentative model illustrating how syndecan-4 may regulate muscle differentiation. During muscle cell proliferation cell surface syndecan-4 function as a co-receptor for FGF2 and its receptor (1), thus increasing their (FGF2 and FGFR) local concentration and leading to enhanced cell growth. At the same time the intracellular domain of syndecan-4 exhibit a negative control of myoblast fusion. Upon differentiation (2) syndecan-4 is internalized from the plasma membrane (3) into endocytic compartments. The recruitment of specific (unknown) adaptor proteins to syndecan-4 enables transport of syndecan-4 from endosomal compartments to the nuclear membrane (4) thus escaping recycling of syndecan-4 back to plasma membrane (5), exosome formation (6) or degradation of syndecan-4 (7). This re-localization may then affect gene transcription and muscle differentiation (8).

In summary, our results add novel and important information to the role of syndecan-4 in muscle differentiation. The role of syndecan-4 in myoblast fusion appears to be complex and does probably both involve interaction with ligands through its extracellular domain and regulation of signalling pathways and intracellular transport through its intracellular domain. How internalization and endosomal sorting of syndecan-4 for transport to the nuclear membrane is regulated is still unclear, and further studies are necessary to address these questions. Likewise is it unclear exactly how the cytoplasmic domain exhibits its inhibiting effect on myoblast fusion. These observations, however, clearly demonstrate that syndecan-4 regulates muscle differentiation at several stages, with different outcomes, both positive and negative, based on interaction with different partners and different cellular localizations. The syndecan family is involved in several signalling pathways, and thus inhibition of syndecan-mediated signalling does often yield different phenotypes [6, 46]. For example, paradoxical results were previously shown upon deletion of syndecan-3, where *Sdc3* ^−/−^ injured muscles, despite a dramatic loss of satellite cells, still were able to retain full regenerative capacity and undergo progressive myofiber size increase over time [46]. We suggest that syndecan-4 at the plasma membrane has a negative effect on muscle differentiation by binding of growth factors, and that internalization of syndecan-4 from the plasma-membrane drives the muscle differentiation according to the following tentative model (Fig 10): Syndecan-4 function as a co-receptor for FGFR and its ligand at the cell surface during proliferation, by increasing their (FGF2 and FGFR) local concentration and thus enhancing muscle cell proliferation. At the same time the intracellular domain of syndecan-4 exhibit a negative control of myoblast fusion. Upon initiation of muscle differentiation syndecan-4 is internalized from the plasma membrane into endocytic compartments. The binding of the syndecan-4, to specific (so far unknown) adaptor proteins, enables transport of syndecan-4 to the nuclear membrane. Syndecan-4 thus escapes recycling or degradation, as well as the localization to exosomes. Our data did not allow us to decide whether the localization of syndecan-4 was restricted to the outer membrane of the nuclear envelope or not. However, the possible transport of syndecan-4 through nuclear pores and localization to the inner membrane of the envelope, where the intracellular domain of syndecan-4 will face the nucleoplasm, may be important in regulation of gene transcription and muscle differentiation.This re-localization of syndecan-4 may affect gene transcription and muscle differentiation. In the future, it will be interesting to study further the molecular mechanisms regulating the various localizations, and the biological functions of syndecan-4, both at the plasma membrane and in the nuclear area, in relation to muscle cell differentiation.
